# The residue of salinomycin in the muscles of olive flounder (*Paralichthys olivaceus*) and black rockfish (*Sebastes Schlegeli*) after oral administration analyzed by LC-Tandem-MS

**DOI:** 10.1186/s12917-023-03867-y

**Published:** 2024-01-12

**Authors:** Seungjin Lee, Won-Sik Woo, Jaekyeong Kim, Yeongwoon Jin, Jin Woo Lee, Jung-Soo Seo, Mun-Gyeong Kwon, Ji-Hoon Lee, Chan-Il Park, Sang Hee Shim

**Affiliations:** 1 Natural Products Research Institute, College of Pharmacy, Seoul National University, Seoul, 08826 Republic of Korea; 2 Department of Marine Biology & Aquaculture, Institute of Marine Industry, College of Marine Science, Gyeongsang National University, Tongyeong, 53064 Republic of Korea; 3 College of Pharmacy, Duksung Women’s University, Seoul, 01369 Republic of Korea; 4 Aquatic Disease Control Division, National Fishery Products Quality Management Service, 337 Haeyang-ro, Yeongdo-gu, Busan, 49111 Republic of Korea

**Keywords:** Veterinary drug, Salinomycin, Residue, Black rockfish, Olive flounder

## Abstract

**Background:**

Salinomycin, an antibiotic, have potential as a veterinary drug for fish due to its anti-parasitic activity against several fish parasites. Thus the residual levels of salinomycin in muscles of two significant aquaculture species in Korea, olive flounder and black rockfish, were analyzed using HPLC-MS-MS.

**Results:**

The proper method to analyze the residual salinomycin in fish muscles using LC-MS-MS was settled and the method was validated according to CODEX guidelines. The residues in three distinct groups for two fish species were analyzed using the matrix match calibration curves at points of five different times following oral administration. After oral administration, salinomycin rapidly breaks down in both olive flounder and black rockfish. After 7^th^ days, the average residue in all groups of two fish spp. decreased below limit of quantitation (LOQ).

**Conclusion:**

Due to low residue levels in fish muscles, salinomycin may therefore be a treatment that is safe for both fish and humans. This result could contribute to establishment of MRL (minimal residual limit) for approval of salinomycin for use in aquaculture.

**Supplementary Information:**

The online version contains supplementary material available at 10.1186/s12917-023-03867-y.

## Introduction

The average growth rate of Korean aquaculture production was 6.4% from 2000 to 2020, making Korea the 6th largest aquaculture country in Asia [1]. Overuse of veterinary medications results in drug accumulation in fish, which can constitute a risk to humans by coming into contact with them through the food chain [2, 3]. The biodegradation of veterinary medications is known to reduce their concentration in fish, although chemical characteristics and biotransformation can cause them to reaccumulate [4]. A practical usage guideline and legal control, such as maximum residue levels (MRLs), must be provided because these medications have the potential to have unforeseen negative consequences at even low concentrations [5, 6].

**Fig. 1:**
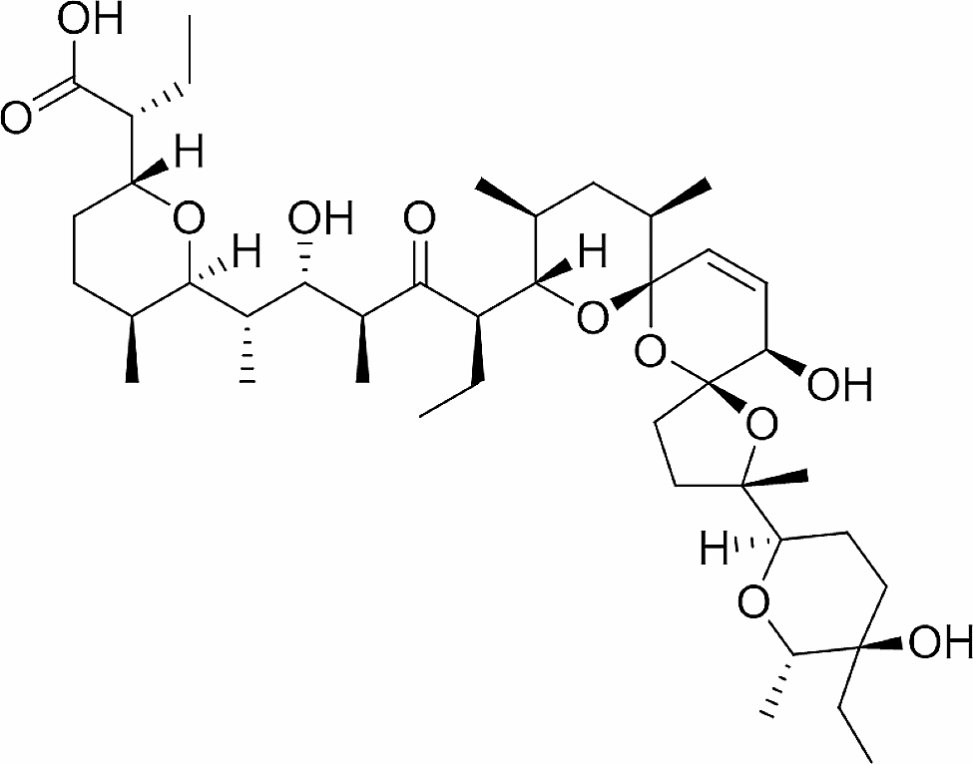
Structure of salinomycin

Salinomycin is one of the natural carboxylic polyether ionophores (Fig. 1), and because of its ability to coordinate with metal cations and transport them into the cellular environment, it exhibits a range of bioactivities [7, 8]. There are several different bioactivities known to exist in it, including antibacterial, antifungal, antiviral, antiparasitic, anticoccidial, and anticancer action [8–10]. Countries such as Canada, the United States, the European Union, Korea, and Japan have approved the use of salinomycin as a veterinary medicine. The specific species that are allowed to use on and the MRLs of salinomycin vary from country to country [11–13]. For instance, MRL for chicken in Japan is at 0.02 mg/kg whereas that for chicken in Korea is 0.1 mg/kg [13]. According to numerous research, salinomycin is particularly effective against fish parasites as *Cryoticaryon irritans*, *Henneguya* sp., and *Ichthyophthirius multifiliis* [14–16]. When fed with a concentration of 200 ppm for two weeks, salinomycin clearly inhibited the development and viability of *C. irritans* with no mortality of *Paralychthys olivaceus* [14]. Salinomycin negatively impacted the plasmodial developmental stage in *Henneguya* sp., and after a 9-day treatment, pansporoblasts were completely destroyed [15]. In an in vivo test, salinomycin completely eliminated *I. multifiliis* at a dosage of 8.0 mg/L [16].

The two most significant aquaculture species in East Asia, including South Korea, Japan, and China, are olive flounder (*Paralichths olivaceus*) and black rockfish (*Sebastes schleglii*), also known as Korean rockfish [17–20]. The production of olive flounder was 41,800 tons, while the output of black rockfish was 17,500 tons in 2021, according to the Korean Statistical Information Service data [21]. This accounts for more than 70% of fish production in Korea. A serious threat to the farming of these two fish species is parasitic disease, particularly *Miamiensis avidus* and *Microcotyle sebastis*. Therefore, it is crucial to develop new antiparasitic agents for fish and/or to investigate the possibilities of extending the use of already-approved veterinary drugs to fish species.

Salinomycin may have potential to be approved as a veterinary medicine for fish due to its respectable antiparasitic activity in fish. The goal of this research is to investigate how much of salinomycin accumulates in the muscles of olive flounder and black rockfish, the two most popular aquaculture fish, and how quickly it disappears to contribute to establish MRL of salinomycin in fish.

## Materials and methods

### Fish samples and treatment of salinomycin

All experimental protocols were performed following the guidelines of the Institutional Animal Care and Use Committee of Gyeongsang National University (approval numbers: GNU-220,519-E0051). 300 olive flounder (*Paralichths olivaceus*), comprising both male and female fish, with an average weight of 70 g were obtained from an aquaculture plant, Yeonhwa susan in Tongyeong, Republic of Korea. Each fish was weighed using a balance. After being put in a 1000 L tank, each fish was allowed a week to adjust. The fish were divided into three groups, designated PO-1, PO-2, and PO-3, at random and each group contains 100 fish. While the water temperature for PO-3 group was maintained at 13.3 ℃, it was retained at 22.3 ℃ for the PO-1 and PO-2 groups. For drug administration, fish was individually weighed and the mixture was administered directly into its gastral cavity using the syringe coupled with an oral zonde; oral doses of 5 mg/kg twice daily for groups PO-1 and PO-3, and oral doses of 10 mg/kg twice daily for group PO-2. To treat fish with the exact dose of salinomycin according to the body weight, we opted for oral administration rather than the more widely used commercial form of therapeutic administration (i.e., dietary exposure). Fish were excluded from the experiment if they regurgitated three minutes after being administered.

Similar to this, 300 black rockfish (*Sebastes schleglii*), male and female combined, with a mean weight of 28.85 g, were bought from an aquaculture plant, Yeonhwa susan in Tongyeong, Republic of Korea. Before administering the medication, all fish spent a week acclimating to their surroundings in a 1500 L tank. Three treatment groups of 100 fish each were randomly assigned the labels SS-1, SS-2, and SS-3. The water temperature for the SS-1 group was fixed at 23 °C, and 5 mg/kg was given orally once per day using zonde. The SS-2 group received 10 mg/kg of oral medication at a water temperature of 23 °C, whereas the SS-3 group received the same treatment at a water temperature of 13 °C. Each group of 15 olive flounder and black rockfish had muscle samples taken on the first, third, seventh, fourteenth, and twenty-eighth days following the last dose. All experimental fish were humanely euthanized using tricaine (TCI, japan) at a concentration of 250 mg/L prior to sampling to ensure a painless procedure. In order to preserve the materials for later analysis, they were frozen and kept at a temperature of -80 °C.

### Sample preparation for analysis

Salinomycin was extracted from the muscles of olive flounder and black rockfish according to the Korean Food Standards designed for animal tissues and announced by the Ministry of Food and Drug Safety [22]. 1 gram of homogenized fish muscle and 1 mL of 0.2 M phosphate buffer were added to the conical tube and vortexed for 1 min. After, 10 mL of acetonitrile was added, vortexed for 5 min, and centrifuged at 4000 rpm for 10 min. The supernatants were collected, completely evaporated, thawed in 1 mL of 50% acetonitrile, filtered with 0.2 μm polytetrafluoroethylene (PTFE) filter, and injected into HPLC-MS/MS system.

### Analysis of residual salinomycin in fish muscle

The concentration of salinomycin in the muscle was assessed through high-performance liquid chromatography HPLC-MS/MS analysis, using Agilent 1260 infinity (Agilent, Santa Clara, CA) and a Thermo LTQ XL (Waltham, MA) linear ion trap mass spectrometer. Nitrogen was used for sheath gas and aux gas. 5 µL of samples were injected into Kinetex^®^ 2.6 μm Polar C_18_ 100 Å LC Column (100 × 2.1 mm) from Phenomenex (Torrance, CA) with a C_18_ resin guard column. The column temperature was set to 30 ℃. Two LC mobile phase solvents (A) 0.1% formic acid solution and (B) 0.1% formic acid acetonitrile were used. The flow rate was 0.3 mL/min, and the total analysis time per sample was 15 min. Solvent composition initially started from (B) 70% and gradually changed to (B) 100% over 8 min. After 3 min of isocratic flow of (B) 100%, solvent composition decreased to (B) 70% in 0.5 min and was eluted with the initial solvent composition for 4.5 min for re-equilibrium. Mass spectrometer acquisition was achieved by selected reaction monitoring (SRM) mode in positive ESI mode with collision energy 58 and 46 (eV). The desolvation gas temperature was adjusted to 320 ℃, and the desolvation gas was Helium.

### Validation of the method

The analysis method used in this investigation was previously developed for use with non-fish animal food products. Therefore, the analysis method was validated in accordance with CODEX guidelines for establishment of a regulatory program for control of veterinary drug residues in food (CAC/GL-16-1993), including linearity, limit of detection (LOD), limit of quantitation (LOQ), accuracy, precision, and repeatability.

Muscles of blank olive flounder and black rockfish were spiked with different concentrations of diluted salinomycin standard stock solution. The stock solution of salinomycin was prepared at a concentration of 10 mg/mL by dissolving accurately weighed 10 mg of salinomycin (analytical grade, Sigma) in 1 mL of methanol. Before spiking, blank samples were tested to confirm that the salinomycin peak was not detected.

The linearity and working range were calculated using a matrix match calibration curve. By injecting the muscle sample with working solution at 6 different concentrations (5 µg/mL, 10 µg/mL, 20 µg/mL, 40 µg/mL, 100 µg/mL, and 200 µg/mL), a linear calibration curve with a coefficient of correlation (*r*^2^) greater than 0.95 was created. Limits of quantitation (LOQ) and limits of detection (LOD) were calculated using the formulas LOQ = 10 / S and LOD = 3.3 / S, respectively, where is the response’s standard deviation and S is the calibration curve’s slope [23]. By comparing the measured area of the spiked samples to the reference sample, a recovery test assessed the accuracy of the analysis procedure. For the precision investigation, three sets of muscle samples (each with five samples) were examined in the same manner on the same day. For the measured concentration, the relative standard deviation (RSD) was determined. By examining 5 measurements of a sample at a certain concentration on 3 distinct days, intermediate precision was put to the test.

## Results

### LC-MS-MS

Based on the method described in the method section, the retention time of the standard salinomycin was consistent at 8.4 min, both within the same day (intra-day) and across different days (inter-day), indicating a decent reproducibility of the analytical method. Ion *m/z* 773.5 [M + Na]^+^ matched to the sodiated form of the molecular ion, and chosen as the precursor ion. Among the product ions, two product ions were used for identification, qualification, and quantitation. The most intense product ion, *m/z* 531.4 and another ion at *m/z* 431.5 was selected as the quantitative ion and qualitative ion correspondingly. All this information was used to confirm that the target component in the sample is identical to the standard substance and does not overlap with other peaks. The retention time of salinomycin in spiked muscle samples also appeared at 8.4 min with precursor ion at *m/z* 773 [M + Na]^+^, quantitative ion at *m/z* 531.4, and qualitative ion at *m/z* 431.3 (Figure S5). Thus, this retention time, precursor ion, quantitative ion, and qualitative ion were used to specifically identify salinomycin by LC-MSMS.

### Method validation

Olive flounder muscle tissue salinomycin HPLC-MS/MS analysis revealed a linear signal magnitude from 5 ng/mL to 200 ng/mL. The limit of detection (LOD) and limit of quantitation (LOQ) were calculated using three calibration curves, which were y_1_ = 6522.04 × _1_ + 9991.27 with *r*^2^ = 0.9997, y_2_ = 6492.71 × _2_ + 11408.9 with *r*^*2*^ = 0.9996, and y_3_ = 657,944 × _3_ + 9017.85 with *r*^*2*^ = 0.9998. The LOD was discovered to be 0.0006 mg/kg and the LOQ to be 0.0018 mg/kg. Salinomycin was found in the muscle of black rockfish, and an HPLC-MS/MS analysis of the sample revealed a linear standard curve with the regression line y = 8087.59x + 19659.1, correlation coefficient of 0.9986, LOD and LOQ values of 0.0032 mg/kg and LOQ, 0.0097 mg/kg.

No sample was diluted since all of the sample concentrations were below the limit of quantitation. Olive flounder recovery varied from 80.2 to 89.0%, whereas black rockfish recovery ranged from 91.1 to 97.1%. Relative standard deviation (RSD) for olive flounder and black rockfish, respectively, ranged from 1.495 to 9.17% and 0.99–7.48% (Table 1). As a result, it was determined that the current validation approach is valid for figuring out the salinomycin concentration in olive flounder and black rockfish.

**Table 1:** Validation parameters of optimized HPLC-MS/MS method

Fish type	Calibration curve	Salinomycin concentration (ng/ml)	recovery (%, n = 3)	Intraday RSD (%, n = 5)	Interday RSD (%, n = 3)
Olive flounder	y = 6522.04 x + 9991.27	5	89.01	1.95	13.1
	y = 6492.71 x + 11408.9	20	82.93	9.17	-
	y = 6579.44 x + 9017.85	40	80.27	1.49	-
Black rockfish	y = 8087.59 x + 19659.1	5	97.1	7.48	10.5
	y = 8201.38 x + 6546.1	20	96.3	0.99	-
	y = 8169.09 x + 4464.11	40	91.1	4.01	-

### Determination of salinomycin residue

Average residues of salinomycin in the muscles of olive flounder and black rockfish after administration of the drug are shown in Table 2. Both olive flounder and black rockfish have a little level of salinomycin in their muscles. After 14 days, the residue in olive flounder drastically decreased and was below the LOQ. For all the groups (SS-1, SS-2, and SS-3) for black rockfish, residue of salinomycin was found under LOQ as early as the first day. Therefore, it was unnecessary to investigate the residue after the seventh day. Olive flounder and black rockfish’s salinomycin residue in the muscle was not significantly affected by temperature or dosage variations.

**Table 2:** Salinomycin residues in olive flounder and black rockfish muscles in different days

Day	Residues (average) in olive flounder (standard deviation)			Residues (average) in black rockfish (standard deviation)		
	PO-1 (5 mg/kg, 22 ℃)	PO-2 (10 mg/kg, 22 ℃)	PO-3 (5 mg/kg, 13 ℃)	SS-1 (5 mg/kg, 22 ℃)	SS-2 (10 mg/kg, 22 ℃)	SS-3 (5 mg/kg, 13 ℃)
1	0.22 (0.13)	0.20(0.06)	0.13(0.07)	<LOQ	<LOQ	<LOQ
3	0.05 (0.05)	0.12(0.08)	0.05(0.04)	<LOQ	<LOQ	<LOQ
7	0.01(0.01)	<LOQ	0.01(0.01)	<LOQ	<LOQ	<LOQ
14	<LOQ	<LOQ	<LOQ	-	-	-
28	<LOQ	<LOQ	<LOQ	-	-	-

(residue unit: mg/kg, -: not tested, dosages of salinomycin and maintenance temperature in tank were indicated in the parenthesis after each group names)

**Fig. 2:**
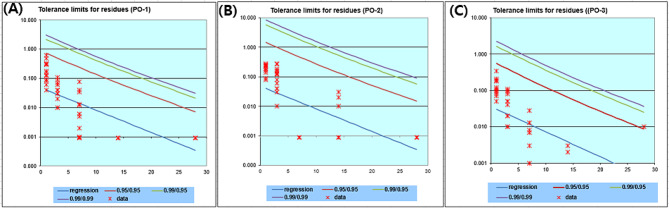
Regression line of salinomycin residue in muscles of olive flounder. (**A**) group PO-1, (**B**) group PO-2, (**C**) group PO-3. The real salinomycin residue of each fish is represented by the red dots (x). The actual residue levels of each fish were used to create the regression line (blue line). The clearance from the 95/95 tolerance limit is shown by the red line; the 99/95 and 99/99 tolerance limits are shown by the green and purple lines, respectively. The “95/95” tolerance limit indicates a 95% level of confidence that the provided limitations are met by 95% of the population, the “99/95” tolerance limit indicates a 99% level of confidence that 99% of the population is also within the limits, and the “99/99” tolerance limit indicates a 99% degree of confidence [24, 25]

The MRL for salinomycin in beef muscle, 0.02 mg/kg, was employed as an interim MRL value because there is no established MRL for salinomycin in fish. According to MRL calculator by JECFA (Joint FAO/WHO Expert Committee on Food Additives), tolerance limit 95% level of confidence that the provided limitations are met by 95% of the population was below the interim MRL, 0.02 mg/kg (for beef muscle) on the third day of drug administration for all six groups. Figure 2 depicts the raw data and regression line for salinomycin residue in olive flounder.

## Conclusion

To track the salinomycin residue over time, the quantitative analysis of salinomycin in the muscles of black rockfish and olive flounder using LC-MS-MS was performed. Salinomycin was extracted from fish muscle in according with the Korean Food Standards notified by the Ministry of Food and Drug Safety. The analytical method performed in this study was validated and found to meet the standards of the CODEX guideline, with *r*^2^ (linearity) over 0.99, and recovery in the boundary of 80–110%, and RSD (relative standard deviation) under 15%.

The outcomes of this study showed that after oral administration, salinomycin rapidly breaks down in both olive flounder and black rockfish. For all three groups in black rockfish, the residue was below LOQ from the first day after drug administration. The residue for olive flounder was equal to or less than 0.01 mg/kg at the seventh day, which is the temporary MRL in Korea in the event that the real MRL is not established. Due to its low residue level in the edible component of fish, salinomycin may therefore be a treatment that is safe for both fish and humans. As shown in the results, the residues of salinomycin in olive flounder were higher than those in black rockfish, indicating that the residue of salinomycin could depend on the fish species. Therefore, it is important to carefully decide to determine the fish species for the residual experiment in order to determine the MRL of salinomycin in fish.

To our knowledge, this study is the first study to analyze the amount of salinomycin left in the muscles of olive flounder and black rockfish after oral treatment. This result could contribute to establishment of MRL for approval of salinomycin for use in aquaculture.

### Electronic supplementary material

Below is the link to the electronic supplementary material.

Supplementary Material 1

## Data Availability

The datasets used and/or analyzed during the current study are available from the corresponding author on reasonable request.

## References

[1] FAO. Aquaculture growth potential in the Republic of Korea. WAPI factsheet to facilitate evidence-based policy-making and sector management in aquaculture. Text by Cai, J., Galli, G., Zhou, In: FAO Aquaculture. Rome, 2023. https://www.fao.org/3/cc4979en/cc4979en.pdf Accessed 21 November 2023.

[2] Zhang H, Chen Q, Niu B (2020). Risk assessment of veterinary drug residues in meat products. Curr Drug Metab.

[3] Chen H, Liu S, Xu X-R, Liu S-S, Zhou G-J, Sun K-F, Zhao J-L, Ying G-G (2015). Antibiotics in typical marine aquaculture farms surrounding Hailing Island, South China: occurrence, bioaccumulation and human dietary exposure. Mar Pollut Bull.

[4] Zhang R, Tang J, Li J, Zheng Q, Liu D, Chen Y, Zou Y, Chen X, Luo C, Zhang G (2013). Antibiotics in the offshore waters of the Bohai Sea and the Yellow Sea in China: occurrence, distribution and ecological risks. Environ Pollut.

[5] Reis-Santos P, Pais M, Duarte B, Caçador I, Freitas A, Vila Pouca AS, Barbosa J, Leston S, Rosa J, Ramos F (2018). Screening of human and veterinary pharmaceuticals in estuarine waters: a baseline assessment for the Tejo estuary. Mar Pollut Bull.

[6] Wang B, Xie K, Lee K (2021). Veterinary drug residues in animal-derived foods: Sample Preparation and Analytical methods. Foods.

[7] Jędrzejczyk M, Janczak J, Huczyński A (2022). Molecular structure and spectroscopic studies of the product of acidic degradation of salinomycin and its potassium salt. J Mol Struct.

[8] Antoszczak M, Huczynski A (2019). Salinomycin and its derivatives - a new class of multiple-targeted magic bullets. Eur J Med Chem.

[9] Antoszczak M, Rutkowski J, Huczyńsk A, Brahmachari G (2015). Structure and biological activity of polyether ionophores and their semisynthetic derivatives. Bioactive natural products: chemistry and biology.

[10] Cheng X, Zheng H, Wang C, Wang X, Fei C, Zhou W, Zhang K (2022). Effects of salinomycin and ethanamizuril on the three microbial communities in vivo and in vitro. Front Microbiol.

[11] Diaz GJ, Aguillón Y, Cortés A (2018). Effects on health, performance, and tissue residues of the ionophore antibiotic salinomycin in finishing broilers (21 to 38 d). Poult Sci.

[12] Chapman HD, Johnson ZB (2002). Use of antibiotics and roxarsone in broiler chickens in the USA: analysis for the years 1995 to 2000. Poult Sci.

[13] Ministry of Food and Drug Safety. The maximum residue limit of veterinary drug in food II-5-17. Korea; 2020.

[14] Yoshinaga T, Im HJ, Nishida S, Ogawa K (2011). In vitro and in vivo efficacies of ionophores against *Cryptocaryon irritans*. Aquaculture.

[15] Dohle A, Schmahl G, Raether W, Schmidt H, Ritter G (2002). Effects of orally administered chemotherapeutics (quinine, salinomycin) against Henneguya sp. Thelohán, 1892 (Myxozoa: Myxobolidae), a gill parasite in the tapir fish Gnathonemus petersii Günther, 1862 (Teleostei). Parasitol Res.

[16] Yao JY, Gao MY, Jia YY, Wu YX, Yin WL, Cao Z, Yang GL, Huang HB, Wang CF, Shen JY (2019). Evaluation of salinomycin isolated from Streptomyces albus JSY-2 against the ciliate, Ichthyophthirius multifiliis. Parasitology.

[17] Hwang HK, Son MH, Myeong JI, Kim CW, Min BH (2014). Effects of stocking density on the cage culture of Korean rockfish (*Sebastes Schlegeli*). Aquaculture.

[18] Nam G-H, Mishra A, Gim J-A, Lee H-E, Jo A, Yoon D, Kim A, Kim W-J, Ahn K, Kim D-H (2018). Gene expression profiles alteration after Infection of virus, bacteria, and parasite in the Olive flounder (Paralichthys olivaceus). Sci Rep.

[19] Niu J, Wang X, Liu P, Liu H, Li R, Li Z, He Y, Qi J (2022). Effects of Cryopreservation on sperm with Cryodiluent in Viviparous Black Rockfish (Sebastes schlegelii). Int J Mol Sci.

[20] Jun JW, Kang JW, Giri SS, Yun S, Kim HJ, Kim SG, Kim SW, Han SJ, Kwon J, Oh WT (2019). Superiority of PLGA microparticle-encapsulated formalin-killed cell vaccine in protecting olive flounder against Streptococcus parauberis. Aquaculture.

[21] Statistics Korea. Results of the year 2022 fish farming trend survey. Text by Department of Agriculture and Fisheries Trends Department, In: Fish farming trend survey. Daejeon., 2023.https://kostat.go.kr/board.es?mid=a10301080400&bid=225&act=view&list_no=424510 Accessed 25 May 2023.

[22] MFDS (2022). Korean Food Standards Codex 8.3.3 Narasin, Laslocid, maduramycin,monesin, salinomycin semduramicin.

[23] ICH. ICH Harmonised Guideline. Validation of Analytical procedures Q2(R1). ICH: International Council for harmonisation of techical requirements for pharmaceuticals for human use; 1995.

[24] Terry L, Kelley K (2012). Sample size planning for composite reliability coefficients: accuracy in parameter estimation via narrow confidence intervals. Br J Math Stat Psychol.

[25] Tan SH, Tan SB (2010). The correct interpretation of confidence intervals. Proc Singap Healthc.

