# Supplementary material for: The residue of salinomycin in the muscles of olive flounder (Paralichthys olivaceus) and black rockfish (Sebastes Schlegeli) after oral administration analyzed by LC-Tandem-MS
Source: BMC Vet Res. 2024 Jan 12;20:24. doi: 10.1186/s12917-023-03867-y (PMC10785415; doi:10.1186/s12917-023-03867-y)
Supplement: Supplementary file 1 — Supplementary Material 1 [file 12917_2023_3867_MOESM1_ESM.docx]

***Supporting Information***

**The residue of salinomycin in the muscles of olive flounder (*Paralichthys olivaceus*) and black rockfish (*Sebastes schlegeli*) after oral administration analyzed by LC-Tandem-MS**

Seungjin Lee,^1+^ Won-Sik Woo,^2+^ Jaekyeong Kim,^1^ Yeongwoon Jin,^1^ Jin Woo Lee,^3^ Jung-Soo Seo,^4^ Mun-Gyeong Kwon,^4^ Ji-Hoon Lee,*^,4^ Chan-Il Park,*^,2^ and Sang Hee Shim*^,1^

^1^Natural Products Research Institute, College of Pharmacy, Seoul National University, Seoul 08826, Republic of Korea
^2^Department of Marine Biology & Aquaculture, Institute of Marine Industry, College of Marine Science, Gyeongsang National University, Tongyeong 53064, Republic of Korea

^3^College of Pharmacy, Duksung Women’s University, Seoul 01369, Republic of Korea

^4^Aquatic Disease Control Division, National Fishery Products Quality Management Service,

337 Haeyang-ro, Yeongdo-gu, Busan, 49111, Republic of Korea

Contents

Figure S1. A diagram of extraction procedure of salinomycin in the muscles of olive flounder and black rockfish.

**Figure S2.** Calibration curves of standard salinomycin spiked in blank muscle sample olive flounder.

**Figure S3.** Calibration curves of standard salinomycin spiked in blank muscle sample of black rockfish.

**Figure S4.** (a) retention time of standard salinomycin (100 ppb), (b) 100 ppb of salinomycin spiked on the muscles of olive flounder, (c) 100 ppb of salinomycin spiked on the muscles of black rockfish, (d) blank muscle.

**Figure S5**. (a) MS-MS fragmentation of standard salinomycin, (b) MS-MS fragmentation of salinomycin spiked on the muscles of olive flounder, (c) MS-MS fragmentation of salinomycin spiked on the muscles of black rockfish.

**Table S1.** Experimental design for analysis of salinomycin residues in olive flounder muscle.

**Table S2.** Experimental design for analysis of salinomycin residues in black rockfish muscle.


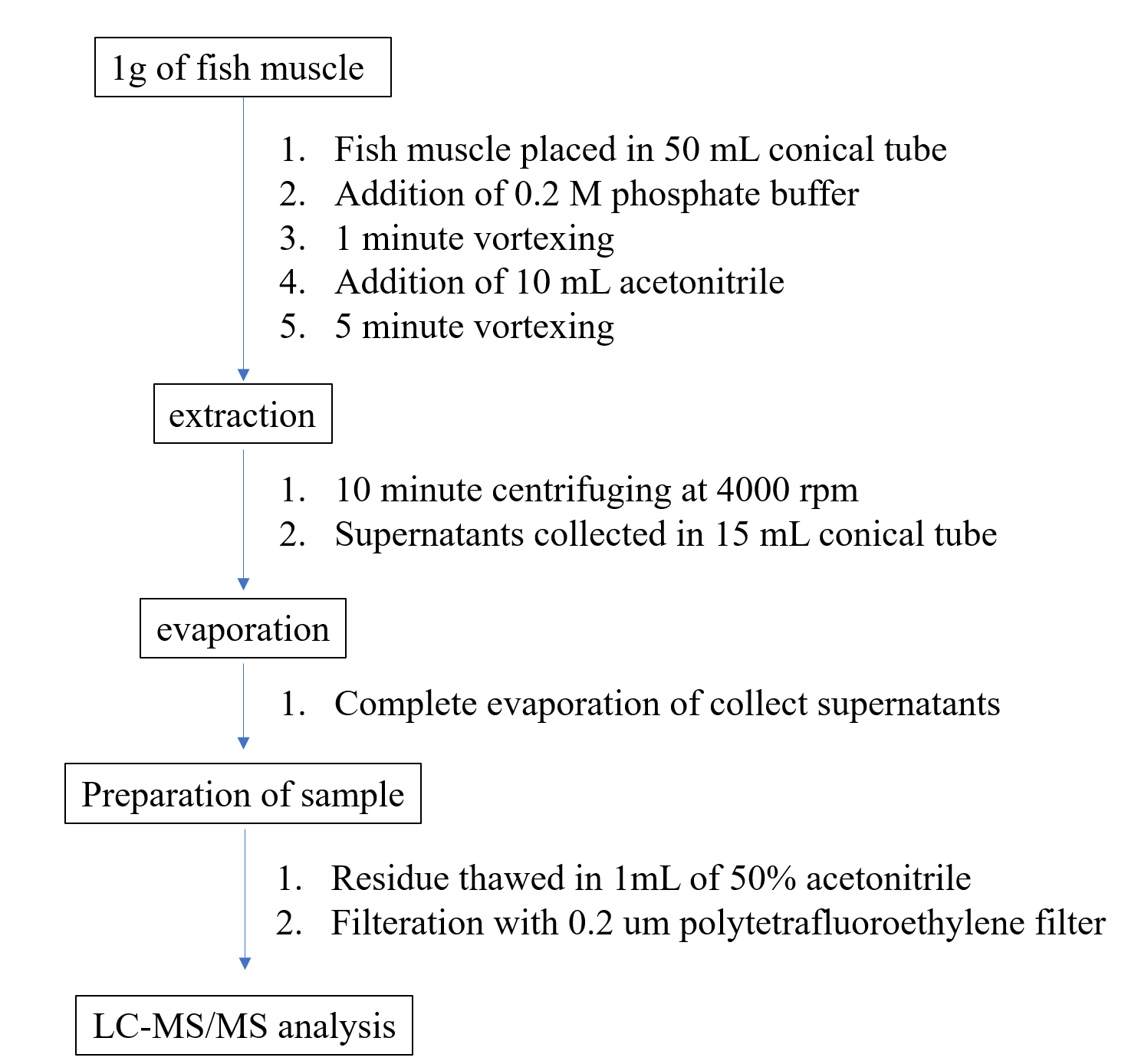


Figure S1. A diagram of extraction procedure of salinomycin in the muscles of olive flounder and black rockfish.


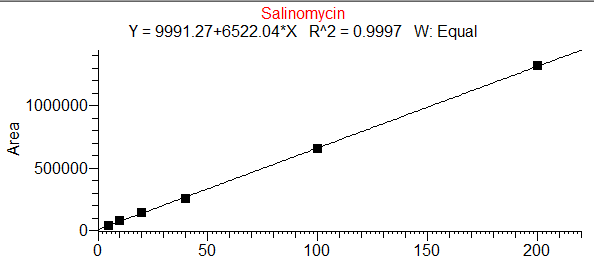
 Y_1_= 6522.04X_1_+9991.27


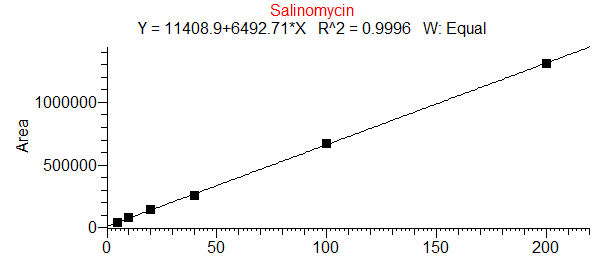
 Y_2_= 6492.71X_2_+11408.9


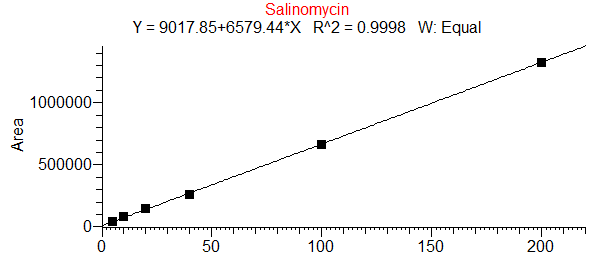
 Y_3_= 6579.44X_3_+9017.85

**Figure S2.** Calibration curves of standard salinomycin spiked in blank muscle sample olive flounder (three independent experiments).


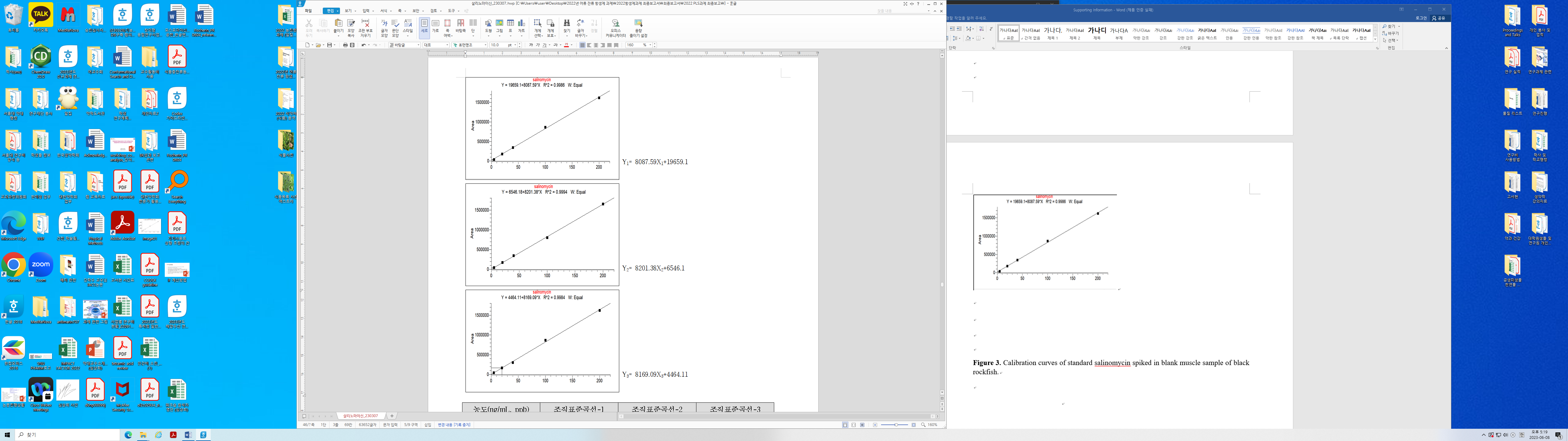


**Figure S3.** Calibration curves of standard salinomycin spiked in blank muscle sample of black rockfish (three independent experiments).

**
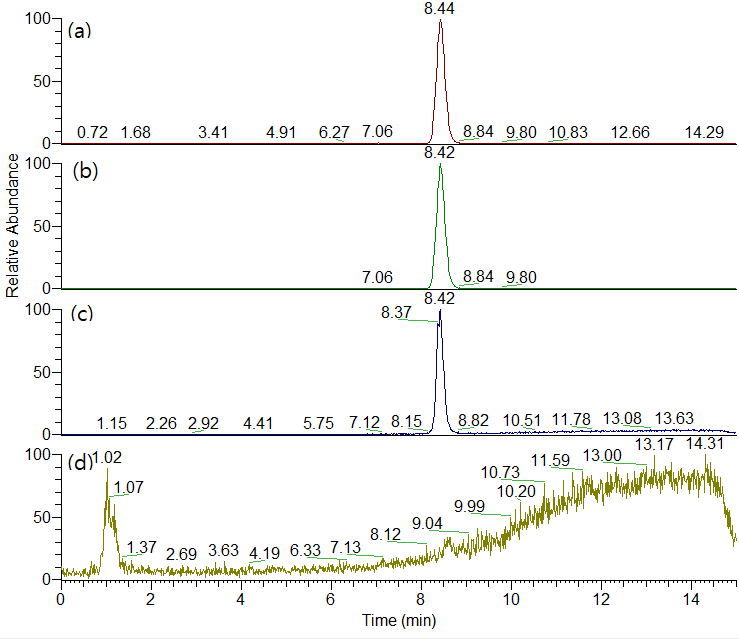
**

Figure S4. (a) retention time of standard salinomycin (100 ppb), (b) 100 ppb of salinomycin spiked on the muscles of olive flounder, (c) 100 ppb of salinomycin spiked on the muscles of black rockfish, (d) blank muscle.


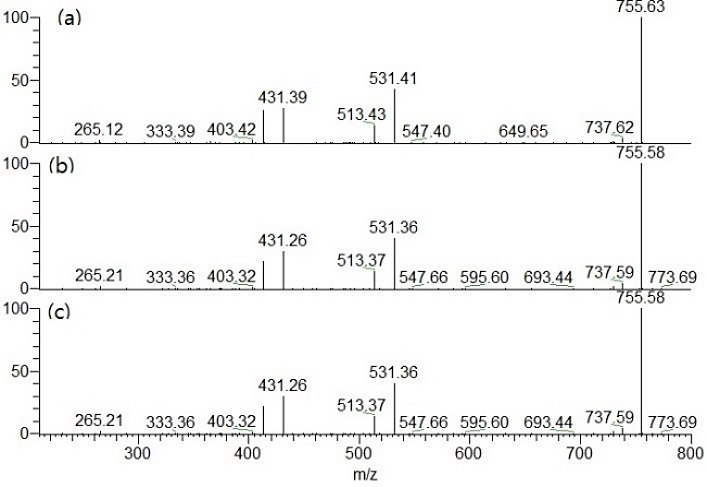


**Figure S5**. (a) MS-MS fragmentation of standard salinomycin, (b) MS-MS fragmentation of salinomycin spiked on the muscles of olive flounder, (c) MS-MS fragmentation of salinomycin spiked on the muscles of black rockfish.

| **Table S1.** Experimental design for analysis of salinomycin residues in olive flounder muscle. | | | |
| --- | --- | --- | --- |
| Treatment group | Water temperature  (± SD) | Dosage | sampling |
| PO-1 | 23±3 ℃ | 5 mg/kg (P.O twice) | day 1, day 3, day 7,  day 14, day 28  (5 sampling total) |
| PO-2 | 23±3 ℃ | 10 mg/kg (P.O twice) |  |
| PO-3 | 13±3 ℃ | 5mg/kg (P.O twice) |  |

| **Table S2.** Experimental design for analysis of salinomycin residues in black rockfish muscle. | | | |
| --- | --- | --- | --- |
| Treatment group | Water temperature  (± SD) | Dosage | sampling |
| SS-1 | 23±3 ℃ | 5 mg/kg (P.O once) | day 1, day 3, day 7,  day 14, day 28  (5 sampling total) |
| SS-2 | 23±3 ℃ | 10 mg/kg (P.O once) |  |
| SS-3 | 13±3 ℃ | 5mg/kg (P.O once) |  |
